# Organoid-based modeling of intestinal development, regeneration, and repair

**DOI:** 10.1038/s41418-020-00665-z

**Published:** 2020-11-18

**Authors:** Joep Sprangers, Irene C. Zaalberg, Madelon M. Maurice

**Affiliations:** 1 Cell Biology, Center for Molecular Medicine, University Medical Center Utrecht, Utrecht, The Netherlands; 2 Oncode Institute, Utrecht, The Netherlands

**Keywords:** Development, Gastrointestinal diseases, Stem-cell research

## Abstract

The intestinal epithelium harbors a remarkable adaptability to undergo injury-induced repair. A key part of the regenerative response is the transient reprogramming of epithelial cells into a fetal-like state, which drives uniform proliferation, tissue remodeling, and subsequent restoration of the homeostatic state. In this review, we discuss how Wnt and YAP signaling pathways control the intestinal repair response and the transitioning of cell states, in comparison with the process of intestinal development. Furthermore, we highlight how organoid-based applications have contributed to the characterization of the mechanistic principles and key players that guide these developmental and regenerative events.

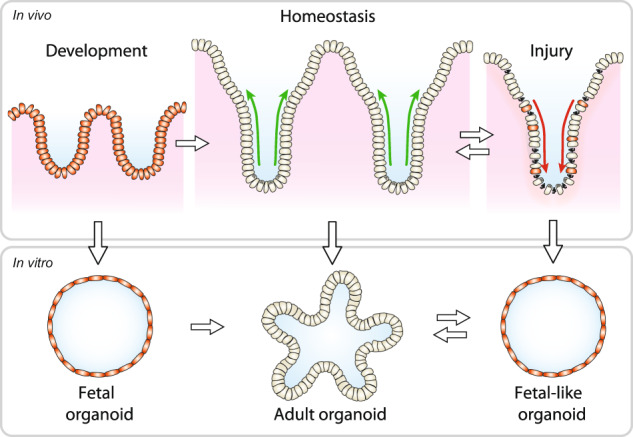

## Facts

- Adult epithelial intestinal cells dedifferentiate into fetal-like progenitor cells upon injury.
- Fetal and adult organoids are faithful models to study the regenerating intestinal epithelium.
- Adult stem cells that fuel homeostatic self-renewal are depleted from the repairing epithelium.
- Intestinal regeneration and repair is guided by alternating roles of YAP and Wnt/β-catenin signaling.

## Open questions

- What damage signals initiate fetal conversion of the intestinal epithelium?
- How do fetal gene signatures correlate with type of injury, reverted cell type, or timing of analysis?
- What is the level of dependency of the regenerative intestinal epithelium on Wnt/RSPO secretion?
- What is the role of β-catenin-independent Wnt signaling during early stages of intestinal regeneration?
- How does the interplay between YAP and Wnt signaling guide the reestablishment of tissue homeostasis after injury?

## Introduction

The intestinal epithelium is a single-cell layer that performs vital functions in food digestion and nutrient absorption and, at the same time, constitutes a barrier against the external environment. Over a lifetime, this barrier is continuously challenged by the harsh environment of the intestinal lumen that contains various microbes, as well as dietary and chemical compounds. Furthermore, therapeutical treatment by ionizing radiation, chemotherapy, or antibiotics may also cause intestinal injury [1]. The intestine has an extraordinary ability to repair itself upon various types of damage. Even radiation doses that almost fully eliminate the proliferative compartment of the intestine in mice can be tolerated. Efficient tissue restoration was shown to involve a phase of apoptosis, to remove damaged cells, and a proliferative burst of surviving cells to replace lost tissue [2]. Over recent years, various studies have indicated that intestinal regeneration and repair depends on the reinitiation of early developmental transcriptional programs in surviving reserve stem cell populations that are quiescent in homeostatic conditions [3, 4]. By reverting to a more primitive state, the intestine thus allows itself to undergo remodeling and induce patterning of newly formed tissue into homeostatic tissue compartments.

Insights in the underlying transient alterations in cell identity and function have greatly benefitted from various applications of organoid technology. Organoids are self-organized, three-dimensional tissue cultures that are derived from stem cells and recapitulate key features of the tissue of origin [5, 6]. As organoids can be directly exposed to injury in vitro and their contribution to tissue repair can be studied by transplantation in mouse models [7, 8], they have provided highly valuable insights into the mechanisms of tissue regeneration as well as the cell types and signaling pathways that drive this process. In this review, we summarize commonalities and differences in the processes of intestinal development and adult intestinal repair, and we highlight how organoid-based applications have contributed to mechanistic insights in the underlying processes. Furthermore, particular focus is placed on the key involvement of Wnt and YAP signaling pathways that guide development, regeneration, and homeostatic tissue renewal.

## Homeostasis of the adult intestine depends on a gradient of Wnt signaling

**Fig. 1:**
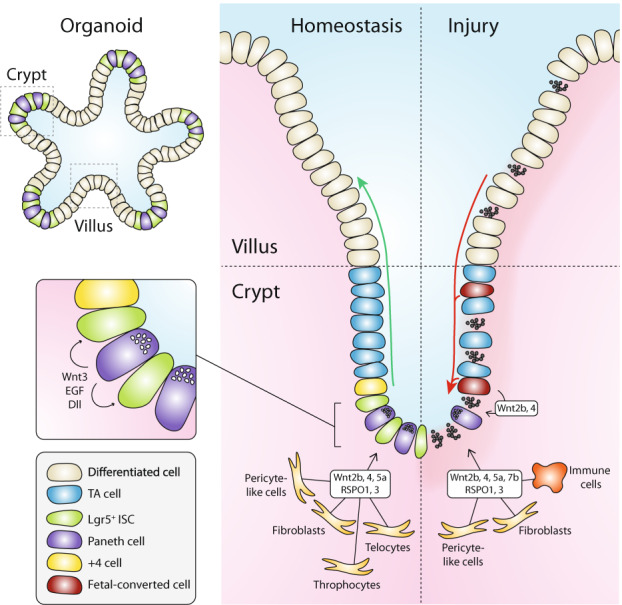
Schematic overview of the intestinal epithelial and mesenchymal cells during homeostasis (left) and injury (right). Schematic organoid is depicted top left. Epithelial and mesenchymal cell types that constitute the stem cell niche are indicated [20–22, 24]. Green arrow represents cell migration from crypt to villus; red arrow represents repopulation of damaged epithelium from fetal-converted reserve stem cells. TA cell transit amplifying cell, ISC intestinal stem cell.

For a detailed overview of the organization of the intestinal epithelium and its cellular constituents, we refer the reader to a number of excellent recent reviews [9, 10]. Briefly, the epithelium of the small intestine is compartmentalized in crypts, that invaginate into the underlying mesenchyme and harbor stem and progenitor cells at their base, and villi, that protrude into the lumen and comprise all differentiated cell types of the secretory, enteroendocrine, and absorptive lineages (Fig. 1). The colonic epithelium lacks villi but is organized similarly, with differentiated cells populating the flattened upper regions of the elongated crypts [11]. The small intestinal and colonic epithelium display a remarkably high rate of self-renewal, which mediates replacement of the epithelium every 4–5 days [12]. The generation of new cells is fueled by *Leucine-rich repeat-containing G-protein coupled receptor (Lgr)5-*expressing adult stem cells that are located at the crypt base[13]. Once Lgr5^+^ daughter cells leave the crypt base, they undergo a brief phase of proliferation within the transit amplifying (TA) zone, while moving upwards along the crypt-villus axis (Fig. 1). Upon exiting the TA zone, these cells will become postmitotic and undergo terminal differentiation, after which they move further upwards to populate the villus region and perform their specialized tasks [14]. Different from this default movement of differentiated lineages, postmitotic Paneth cells will migrate downwards to the crypt base to constitute a specialized niche for the maintenance of Lgr5^+^ stem cells [6]. Just above the crypt base, an alternative stem cell pool is located at the so-called +4 position (Fig. 1). These cells were identified as slow-cycling cells that are fated toward the secretory lineage [11]. While +4 cells have been signified by a variety of markers, a shared feature is their pivotal role during injury-induced epithelial regeneration [4], as discussed below. During homeostasis, the proliferative activity of Lgr5^+^ stem cells is controlled by neighboring Paneth cells as well as specialized mesenchymal cells that reside in close proximity of the crypt base [15]. Collectively, these cells constitute the stem cell niche that provide essential signaling factors, including various Wnts, as well as epidermal growth factor, Notch, and bone morphogenic protein (BMP) inhibitors (Fig. 1) [9].

Wnt/β-catenin signaling mediates a critical role in stem cell maintenance by forming a gradient that peaks at the base of the crypt [16] (Fig. 1). Wnt/β-catenin signaling is initiated by binding of Wnt family members to their receptors at the cell surface, comprised of the Frizzled (FZD) family and the coreceptors low-density lipoprotein receptor-related protein (LRP) 5 or 6 [17]. Wnt-mediated receptor activation leads to the inactivation of the cytosolic β-catenin destruction complex, which induces the stabilization and nuclear entry of the transcriptional co-activator β-catenin and the expression of Wnt target genes [18]. To secure high levels of Wnt signaling within the niche, Wnts are provided by multiple sources (Fig. 1). Paneth cells provide an epithelial source of Wnt3 that remains cell-bound and signals via direct cell–cell contact to neighboring stem cells [16]. In addition, various other Wnts produced by mesenchymal cells were found to sustain stem cell maintenance when epithelial Wnt secretion is perturbed, suggesting redundant functions of both niche components [19–24]. Importantly, mesenchymal niche cells also secrete proteins of the R-spondin (RSPO) family [22, 25, 26], that are highly potent amplifiers of epithelial Wnt signaling (Fig. 1) [27]. Taken together, high local levels of Wnt signaling are critical for the maintenance of intestinal stem cells and homeostatic self-renewal. This observation is further substantiated by the usage of Wnt target genes, including *Lgr5*, *Rnf43*, and *Axin2*, as bona fide markers for identification of intestinal adult stem cells [13, 28, 29].

## Intestinal development

### Alterations in epithelial morphology and organization guide intestinal development

**Fig. 2:**
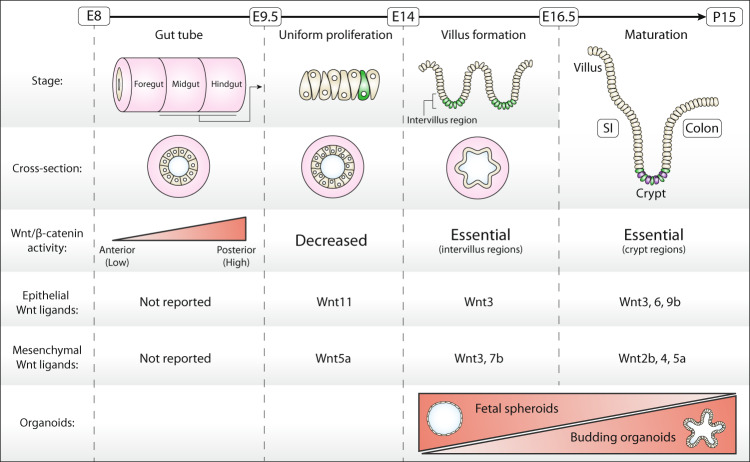
Stages of intestinal development. Intestinal development can roughly be divided into four stages: (1) gut tube formation and patterning, (2) uniform proliferation and growth, (3) villogenesis, and (4) maturation. Schematic representation of the intestinal epithelium, gut tube cross-sections, Wnt/β-catenin signaling activity, expressed Wnts (epithelium and mesenchyme), and organoid phenotypes are depicted. Green cells represent Lgr5^+^ intestinal stem cells (ISC). SI small intestine.

Early gastrointestinal development in mice starts at embryonic day (E)8 after fertilization. From E8 to E9.5, the gut tube is formed and patterned into the foregut, midgut, and hindgut regions [30]. At the same time, these regions develop and specialize into organ-specific domains of the adult animal. While the foregut gives rise to esophagus, lungs, stomach, liver. and pancreas, the intestine develops from the mid- and hindgut [30]. Next, between E9.5–E14, the gut tube is shaped into a pseudostratified layer characterized by uniform proliferation [31]. As a result, the intestine rapidly increases in length, circumference, and luminal area (Fig. 2).

Starting from E14, the pseudostratified intestine changes morphology to a simple columnar epithelium [32], which, from E15, is patterned into villus and intervillus regions that emerge in a proximal-to-distal wave [32, 33]. At this stage, proliferation becomes largely confined to the continuous intervillus regions that precede crypt morphogenesis [34–36]. From E16.5 on, the intestinal epithelium undergoes further maturation, as illustrated by the gradual formation of crypt-villus structures comparable to those in adult mice [35, 37]. At the same time, precursor cells begin to differentiate into the various cell types that populate the adult intestine, including Goblet cells, enteroendocrine cells, and absorptive enterocytes (small intestine) or colonocytes (colon) [32]. Of note, in mice this process continues until 2 weeks after birth, while human intestinal development is completed before birth [38]. During this final maturation phase, intestinal elongation is further promoted by both crypt and villus fission events [34, 39–41]. Although the frequency of crypt fission events is higher during development, this process still occurs at low frequency in the adult epithelium as well [39, 42].

### Fetal organoids retain early developmental features

Over the past decade, fetal intestinal organoid cultures have emerged as useful models for intestinal development [36, 43]. Successful derivation of organoids from fetal intestines was described for various developmental stages, ranging from E14 until early postnatal life (postnatal day P15) for mice [34, 36, 43, 44] and gestational weeks 10–22.5 for human fetal tissue [36, 45]. Unlike adult mouse small intestinal organoids, fetal gut in vitro cultures commonly yield a mixture of spheroid-shaped and budding organoids, in which budded structures represent crypt regions carrying stem and Paneth cells. The ratio between these two phenotypes changes with developmental stage, as shown by a progressive increase in the fraction of budding organoids for cells isolated at later stages [36, 43]. Furthermore, transcriptional profiles of human fetal organoids isolated at different timepoints during development display a progressive similarity to those of adult organoids [45]. These observations indicate that the identity of fetal organoids is linked to their developmental phase of culture derivation. A noteworthy exception is mouse intestinal organoids derived from late stages of fetal development (E19). When cultured in vitro, these late fetal intestinal organoids undergo spontaneous epithelial maturation from spheroids carrying neonatal markers to budding organoids that express hallmarks of the adult epithelium [46]. Thus, at later stages in development, epithelial maturation appears intrinsically programmed and not depending on microenvironment, dietary, or hormonal signals.

### Stemness properties are malleable during development

The utilization of fetal organoids has greatly contributed to the characterization of early intestinal progenitors as well as the molecular alterations that underlie the transition of the epithelium into a mature, homeostatic adult state. Contrary to adult small intestinal organoids, in which proliferation is confined to crypt-like budding structures, fetal spheroids show a uniform proliferation pattern [36] (Fig. 2). Moreover, organoid transcriptomes at fetal and adult states reveal profound differences in cellular composition and stemness properties. Most prominently, E14-derived fetal spheroids display a lack of adult intestinal stem cell markers (*Lgr5*, *Smoc2*, *Axin2*, *Cdx1*, *Tert*, and *Olfm4*) and an absence of differentiated lineages such as enterocytes, Goblet, enteroendocrine, and Paneth cells [36, 43]. Instead, these spheroids show prominent expression of a fetal transcriptional program, marked by high levels of *Trop2* and *Cnx43* that are also highly expressed in the E14 intestine [43].

In line with these findings, the ability to grow budding organoids correlates with the presence of cells expressing adult stem cell markers (*Lgr5*, *Ascl2*, and *Olfm4*) that appear from E15.5 in the proliferative intervillus regions of the developing intestine [47–49]. Indeed, Lgr5-GFP^+^ reporter cells isolated from E15.5 or P2 neonatal intestinal epithelial tissue readily formed budding organoids, while isolated Lgr5-GFP^−^ cells mainly gave rise to spheroids [5, 36, 44]. In addition, diphtheria toxin-mediated depletion of Lgr5^+^ cells [50] from established fetal organoid cultures severely compromised growth of budding organoids, while spheroids remained unaffected [43]. Thus, the ability of organoids to self-organize in crypt and villus domains is strongly linked to the presence of Lgr5-expressing cells.

Although Lgr5^+^ cells detected in the intervillus regions of the E15.5–16.5 intestinal epithelium were found to give rise to adult intestinal stem cell populations, the expansion of Lgr5^+^ cell progeny alone is not sufficient to sustain growth of the fetal epithelium [33, 34, 44]. In fact, transplanted spheroid cultures derived from both Lgr5^+^ and Lgr5^−^ cells isolated from E16.5 intervillus and villus regions were equally potent in regenerating the mouse intestinal epithelium in vivo after induction of colitis [34]. Moreover, during the process of “villification” in which villi undergo prominent fission events, E16.5 cells were found to switch reversibly between Lgr5^+^ and Lgr5^−^ states, depending on cell position. Together, these findings indicate that cell fate potential in the fetal, developing epithelium is not hardwired but rather represents a general intermediate state during tissue maturation.

### Roles of Wnt signaling in the developing intestinal epithelium

In the adult intestinal epithelium, a Wnt gradient that peaks at the base of the crypt is required to maintain Lgr5^+^ stem cell populations and drive progenitor proliferation (Fig. 1). For the developing intestine, the role of Wnt signaling is much less defined. During early gut formation (E8–E9.5), an increasing anterior-to-posterior Wnt/β-catenin gradient regulates patterning of the gut tube [31, 51]. At these early stages, Wnt/β-catenin signaling in the mid- and hindgut induces expression of the transcription factor Cdx2, a key mediator of intestinal identity [51–53].

In the next stage (pre-villus; E9-E14), marked by uniform proliferation, Wnt/β-catenin signaling appears reduced. Along with the low expression of canonical Wnt target genes (e.g. *Ascl2, Axin2 and Rnf43*), the expression of negative regulators such as soluble FZD-related proteins is upregulated [44, 54, 55]. Moreover, mice bearing an epithelial-specific deletion of β-catenin or a germline deletion of β-catenin co-factor *Tcf4* do not show a phenotype in the small intestine before onset of villogenesis [47, 54]. Also, Wnt reporter (TOP-GAL) activity becomes apparent only from stage E15.5, when Lgr5^+^ cells have emerged [56]. Thus, multiple lines of evidence indicate that Wnt/β-catenin signaling does not sustain the uniform proliferative state of the epithelium.

Despite the general decrease in Wnt/β-catenin activity, expression of *Wnt5a* (mesenchymal) and *Wnt11* (epithelial) that are mainly linked to alternative, β-catenin-independent pathways of Wnt signaling, is strongly increased between E10 and E14 [54, 57–59]. Germline *Wnt5a* deletion leads to shortening of the gut, indicating a role of Wnt/β-catenin-independent signaling in intestinal elongation [57, 60]. In agreement, epithelium-specific deletion of *Wntless*, a key protein involved in Wnt trafficking and secretion, does not affect proliferation, while its removal from the mesenchyme leads to significant gut shortening at E13.5 [54]. As an underlying mechanism, a recent study showed that after mitotic division is completed at the apical side of the pseudostratified epithelium, mesenchymal Wnt5a guides the outgrowth of a “pathfinding” filopodium that mediates attachment and subsequent migration of the nucleus to the basal side. In absence of Wnt5a, cells fail to tether basally and are lost by apoptosis, leading to a shortened gut [59].

Even though overall Wnt/β-catenin signaling is low or absent in the uniform proliferation phase, Lgr5^+^ cells start to appear from E12.5 onwards in a distal-to-proximal wave, thus occurring before the onset of villus formation [33, 44]. These early Lgr5^+^ cells express low levels of Wnt/β-catenin target genes and their transcriptional program only partially overlaps with those of adult Lgr5^+^ intestinal stem cells [44]. The appearance of early Lgr5^+^ cells is regulated by the transcriptional regulator Id2 that prevents premature Lgr5 expression [44]. Strikingly, deletion of *Id2* mediates increased expression of *Wnt6* and *Wnt11* in the developing intestine, and treatment with the highly potent Wnt secretion inhibitor C59 prevents early Lgr5 expression at E11.5 in *Id2*-knockout mice. Moreover, C59 treatment also inhibits *Lgr5* expression during gut elongation in wild-type mice, further stressing a role for Wnt-mediated signaling in the emergence of Lgr5^+^ cells. Thus, despite observations of an overall reduction in Wnt/β-catenin signaling, the timed regulation of Wnt activity by Id2 controls the appearance of early Lgr5^+^ cells.

Once villus morphogenesis is initiated, Wnt/β-catenin signaling is reactivated by a poorly understood mechanism that likely involves induced expression of epithelial and mesenchymal *Wnt3* as well as mesenchymal *Wnt7b* [54]. The formation of a Wnt/β-catenin signaling gradient is promoted subsequently via an intricate interplay between mesenchyme and epithelium that controls epithelial invagination and villus formation [31]. During this process, epithelial buckling forces create pockets under the villus tips that mediate a local increase in the concentration of the secreted morphogen Sonic Hedgehog (Shh). This promotes BMP secretion by the mesenchyme which locally dampens Wnt signaling [33, 61]. Thus, these combined events help to establish a gradient of Wnt signaling which restricts the location of proliferating cells to intervillus regions. At the same time, intestinal stem cell identity is gradually enforced by the increased expression of Wnt target genes such as *Lgr5*, *Axin2*, and *Cd44* [44, 54]. The key importance of Wnt/β-catenin signaling at this stage is shown by the severe effects of deletion of Wnt pathway components including *β-catenin*, *Tcf4*, or *Lrp5/6* on intestinal proliferation and villus morphogenesis [47, 54, 62].

After birth, crypts are starting to form in the mouse intestinal epithelium by forces generated by myosin-II dependent apical constriction, local differences in matrix stiffness as well as nonuniform proliferation [35, 63]. These events are maintained in budding organoids, indicating that these properties are intrinsic to the self-organizing intestinal epithelium [64]. By P13, *Lgr5* expression has shifted from the intervillus region to stem cells at the base of mature crypts [35, 65]. At the same time, *Lgr5* expression levels increase, while the total number of Lgr5^+^ cells decreases during crypt development, indicating that Lgr5^+^ cells undergo maturation [35]. These features of crypt development are also observed in maturing organoids [46, 66]. Strikingly, mature Paneth cells only appear 2 weeks after birth when crypt development is finalized [39, 67–69]. This comprises the last step in the maturation process to establish a homeostatic self-renewing intestinal epithelium.

### Fetal spheroids display a differential requirement for Wnt and R-spondin

While budding organoids derived from the intestine of E14.5–E16.5 embryos display a clear dependency on Wnt for growth [36, 43], for fetal spheroids the picture is less clear. In one study, blocking Wnt secretion by treatment of spheroids with Porcupine inhibitor IWP-2 for 4 days resulted in a dose-dependent reduction of growth, while Wnt3a supplementation could rescue these effects [43]. In addition, RSPO-mediated Wnt potentiation was required for E15.5 and E16.5 fetal spheroid survival [43, 44]. RSPO-mediated signaling in the intestine depends on expression of the paralogues *Lgr4* and *Lgr5*, of which the former is expressed in all crypt cells while *Lgr5* is expressed exclusively in stem cells [27, 70]. Strikingly, while *Lgr5* deletion does not affect small intestinal epithelium development, *Lgr4* knockout mice lack fetal Lgr5^+^ progenitors and show impaired proliferation at E16.5 [48]. Furthermore, Lgr4 was found essential for fetal spheroid and fetal organoid growth [43, 70, 71]. By contrast, another study reported that treatment of fetal spheroids with IWP-2 was well-tolerated and, moreover, was found to drive an increase in the spheroid/organoid ratio [36]. In accordance, RSPO supplementation was also not required in this study and, furthermore, supplementation with exogenous Wnt3a mediated maturation of spheroids to organoids [34, 36]. Thus, although most studies indicate that fetal spheroid growth depends on epithelial Wnt production and RSPO/Lgr4-mediated Wnt potentiation, this phenotype might not be shared by all fetal spheroid cultures. Potentially, the differential requirement for growth factors may reflect the transitional stage of the intestinal epithelium in which cells that display Wnt-independent and Wnt-dependent growth states may coexist. The mechanism behind this heterogeneity awaits further investigation.

## Intestinal regeneration

### The intestinal injury response involves a program of epithelial dedifferentiation

To preserve integrity of the epithelial layer and prevent infections, the intestine has developed a remarkable ability to sense damage and undergo regeneration and repair (Fig. 1). An increasingly detailed picture of the changes in cellular activity that occur during intestinal damage repair has emerged from a variety of mouse models that involve endoscopy-guided mucosal wounding [72], parasitic infection [73], irradiation [74], dextran sulfate sodium (DSS)-induced colitis [75] as well as targeted methods for adult stem cell ablation [50].

In general, the sensing and repairing of various types of intestinal damage is divided in an injury phase marked by damage sensing and acute control, a regenerative phase characterized by a burst of proliferation, and a normalization phase in which homeostasis is restored. A first response to mucosal wounding is the mobilization of non-proliferative cells from neighboring crypts termed wound-associated epithelial (WAE) cells that migrate over the wound bed in an acute response to maintain barrier function, a process called restitution [72, 76]. In case of irradiation damage, the first phase is characterized by a significant level of apoptosis during which crypts shrink in size or are completely lost [77]. This acute injury phase is followed by a phase of intense proliferation in which surviving crypt (reserve) stem cells proliferate into large regenerating crypts [74, 78]. This phase generally lasts until 4 days after injury [77]. Next, a normalization phase follows until day 7 post injury, in which crypt size and number are restored.

Emerging evidence indicates that regeneration of the damaged intestine requires the transient reprogramming of the epithelium into a fetal-like state [73, 75, 79]. Guided by an incompletely understood crosstalk between the damaged epithelium, the underlying mesenchyme and recruited inflammatory cells, the epithelium is induced to undergo a program of dedifferentiation characterized by the suppression of markers for adult stem and differentiated cells and the de novo expression of a fetal gene signature. Importantly, stem cell potential is fully retained within this regenerative state. Indeed, a collection of lineage tracing studies indicates that various sources of cells can revert to a stem-like identity following the loss of crypts. For a detailed discussion, we refer the reader to a number of excellent recent reviews [3, 4, 80]. In summary, a variety of cell types, including progenitors of the secretory and absorptive lineages located at the “+4 position” as well as differentiated Paneth and enteroendocrine cells, were found to act as reserve stem cell pools that become reactivated upon damage, contribute to the regenerative response, and help re-establish homeostasis by restoring the Lgr5^+^ stem cell pool and establishing a crypt-villus epithelial organization [81–84]. A shared and required factor for reserve stem cell capacity may be the location of cells in close vicinity of the crypt, where niche signals are found [79, 85]. Collectively, these findings signify a remarkable plasticity of the intestinal epithelium, which involves reinitiation of fetal transcriptional programs that allow the intestinal epithelium to remodel itself and sustain function after injury.

### Modeling epithelial regeneration using organoids

**Fig. 3:**
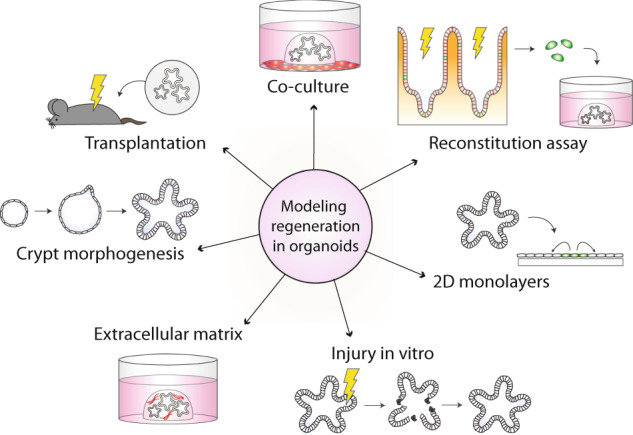
Modeling regeneration in organoids. Overview of various organoid-based models and applications to study aspects of intestinal regeneration.

Organoid-based applications have been instrumental in the characterization and functional analysis of the damage-induced regenerative intestinal epithelium, in various ways (Fig. 3). To study growth properties of the repairing epithelium in vitro, cells of damaged areas expressing the fetal marker Sca-1 were isolated from the small intestine of helminth-infected mice [73] or the inflamed colon of DSS-treated mice [75] and cultured under standard organoid conditions. Whereas control Sca-1^−^ cells isolated from helminth-infected mice formed typical budding organoids, Sca-1^+^ cells generated smooth spheroids that expressed various fetal markers, lacked budding structures and could be stably passaged [73]. Furthermore, Sca-1^+^ spheroids were deficient of Wnt target gene expression and markers of differentiation and their growth was insensitive to RSPO withdrawal, displaying similarity to fetal spheroids [36, 73]. Thus, despite the transient nature of the fetal-like reprogramming of the damaged epithelium in vivo, this reacquired primitive state can be maintained and propagated in vitro using organoid-based culture protocols. Additionally, the stem cell capacity of fetal-reprogrammed colon of DSS-treated mice was confirmed by reconstitution assays, in which isolated Sca-1^+^ cells grown in vitro displayed a spheroid appearance [75]. Moreover, in vitro grown fetal-like spheroids carried the capacity to regenerate differentiated cell types of the intestinal epithelium, as shown by orthotopic transplantation experiments in mice with DSS-induced colitis [75]. These experiments also indicate that the propagated fetal-like state of in vitro cultured regenerative spheroids is reversible, thus supporting a model in which the epithelium is capable to transition between cellular states.

Epithelial switching between injury, repair, and homeostatic states was modeled recently in vitro by growing colonic spheroids in a 2D layer in an air-liquid transwell system supplemented with standard organoid medium [86]. By exposing epithelial cultures to an air-liquid interface during 21 days, the colonic epithelium acquired a columnar shape and exhibited homeostatic self-organization, with alternating proliferative and differentiated regions. Submerging these cultures induced epithelial damage marked by hypoxic injury and ER stress, representing established features of colitis [86]. Reexposure to the air-liquid interface initiated a regenerative state marked by a proliferative burst of Hopx^+^ fetal-like stem cells, after which homeostasis was restored [86]. Of note, full restoration of homeostasis may not be achieved in this model system, as Lgr5^+^ stem cells were not identified. Recently, another study employed tubular-shaped hydrogels engrafted with Lgr5^+^ cells to grow mini-guts in vitro with a perfusable lumen. This set-up was shown to accurately model injury and repair phases of epithelial wounds, radiation injury, and DSS-induced damage [87]. In addition, direct irradiation of cultured organoids may also mimic aspects of in vivo regeneration, as revealed by an initial loss of budding structures and stem cell marker expression, and a gradual recovery over 96 h after radiation exposure [88, 89]. Besides, in vitro radiation damage of organoids may be helpful to assess plasticity of multiple differentiated lineages [90, 91]. Together, these studies indicate that in vitro epithelial cultures may accurately recapitulate cycles of injury and repair, providing exciting opportunities for an in-depth investigation of the molecular basis of epithelial adaptability.

### Alternating roles of YAP and Wnt signaling control epithelial regeneration

Along with the induction of a fetal-like transcriptional program, the intestinal epithelium is transiently depleted of Wnt-driven Lgr5^+^ stem cells and associated Paneth cells following several types of injury [73, 75, 79, 92]. Together, these features are highly reminiscent of the uniform proliferation phase of the developing intestine, underscoring the view that an early developmental program is employed to remodel and repair the epithelium and restore homeostasis. The drop in Wnt signaling activity comprises an early epithelial damage response that is rapidly reverted, as *Lgr5* and *Olfm4* expression is recovered at 3–5 days after injury [73, 79, 92].

Multiple studies have suggested that a transient increase in YAP signaling, a major mechanosensing pathway [93], is responsible for the decreased activity of Wnt signaling in the regenerative epithelium. First, the intestinal epithelial gene program induced by activated YAP signaling revealed significant overlap with the gene signature of fetal intestinal spheroids [66, 75, 79, 94]. Furthermore, YAP and TAZ, key transcriptional coregulators of the YAP pathway, were found associated with the β-catenin destruction complex. Moreover, their nuclear entry and activity was shown to depend on Wnt activity, indicating that both pathways are intertwined [95]. In another study, a mechanistic model was proposed in which Wnt5a/b-mediated signaling directly activates nuclear YAP to antagonize Wnt/β-catenin signaling [96]. Indeed, deletion of YAP from small intestinal organoids promoted the expression of Wnt target genes, including *Lgr5* and *Axin2*, and mediated downregulation of genes associated with the regenerative response [92]. By contrast, overexpression of YAP blocked the expression of Wnt target genes in organoids [94] and inhibited proliferation and crypt formation [92]. Lastly, deletion of YAP from the intestinal epithelium in vivo mediated sustained Wnt signaling in an early phase of injury [92].

A recent study sheds light on how intestinal damage mediates regenerative YAP activation [97]. Prostaglandin E2 (PGE2), produced by a rare subset of PDGFRα^low^ fibroblasts, was identified as a key mediator of signaling between the mesenchyme and intestinal epithelium upon irradiation damage. Indeed, organoids grown in the presence of PGE2 transitioned into spheroids, while organoids lacking Ptger2, the receptor for PGE2, failed to do so. Moreover, PGE2-Ptger2 signaling between fibroblasts and epithelium drove the expansion of Sca-1^+^ reserve stem cells, that displayed reduced expression of β-catenin target genes but an increased YAP-target signature [98]. These findings are in line with an earlier study that identified PGE2 as a key mediator of the expansion of transient repair (WAE) cells upon intestinal wounding [99]. Thus, PGE2-Ptger2 signaling promotes YAP-mediated regeneration of the epithelium.

An important challenge will be the understanding of how the regenerative epithelium switches from active YAP signaling to normalization of tissue homeostasis by re-establishing Wnt-high crypt regions that create a niche for Lgr5^+^ adult stem cells. A first mechanistic insight in this process may have come from a recent study in which intestinal organoids were followed during their growth and development in vitro [66]. At early stages after seeding, organoids adopted a regenerative state showing a fetal signature and high uniform nuclear YAP expression. At later stages when organoids had grown to 8- or 16-cell size, cell-to-cell variability in YAP expression occurred. Next, this symmetry-breaking event led to the formation of the first Paneth cells via Notch-mediated lateral inhibition [66]. Thus, it will be interesting to investigate whether the switching of YAP-active to YAP-inactive states may represent a key event to initiate the normalization phase of injury repair in vivo.

Taken together, the intestinal epithelial injury response is characterized by an immediate and transient decrease in the activity of Wnt/β-catenin signaling and a concomitant upregulation of YAP signaling to drive the temporary expansion of reserve stem cells to initiate tissue regeneration.

### Wnt signaling is required for intestinal regeneration and repair

Paradoxically, even though Wnt/β-catenin signaling appears suppressed during the first days after injury, a wealth of evidence indicates that the expression of Wnt signaling components is essential during intestinal injury repair. In various mouse models of intestinal damage, including irradiation, DSS- and trinitrobenzene sulfonic acid (TNBS)-induced colitis, mucosal wounding, as well as bacterial and viral infection, the expression of Wnt ligands was induced within the first days after injury (summarized in Table 1). Recurrent Wnts that are induced upon intestinal damage are Wnt2b, 4, 5a, and 7b and expression involves both epithelium and mesenchyme [24, 72, 78, 100, 101]. The induction of Wnt expression is accompanied by the expression of RSPO1 and 3 in various cases [20, 26, 102]. In addition, exposure to 12Gy gamma-irradiation induced Wnt/β-catenin signaling in the intestine, as measured in Wnt reporter (TOP-GAL) mice [103].

**Table 1:** Overview of increased Wnt and RSPO expression during various injury models.

Damage type	Dose	Timing	Epithelial Wnt/RSPO	Stromal Wnt/RSPO	References
Irradiation	WBI, 10 Gy (in vivo) or 8 Gy (in vitro)	24 h	Wnt2b, 4 (crypt)	Wnt7b	[78]
	WBI, 9 Gy	48 h		Wnt2b, 4 (Ng2^+^)	[24]
	WBI, 12 Gy	24 h	Wnt3, 6, 9b		[103]
DSS-induced colitis	3.5% DSS	7 days		Wnt3a, 5b, 10a (MPs and DCs) Wnt2, 8a, 8b, 11 (MPs)	[112]
	2.5% DSS	3 and 5 days		RSPO3 (Gli1^+^ cells)	[20]
	2.5% (acute) 2% (chronic)	7 days 2 cycles of 7 days		RSPO1 (CD34^+^ cells) Wnt5a (MyoF)	[26]
	2.5%	7 and 10 days		Wnt5a	[101]
	1.5–2.5%	3 and 7 days		RSPO3 (Myh11^+^ MyoF)	[102]
TNBS-induced colitis	3.5 mg per 20 g body weight	4 days	Wnt2b	Wnt2b, 7b, 10a (MPs)	[100]
Mucosal wound	NA	4 and 6 days		Wnt5a	[72]

Irradiation data from small intestine, chemical colitis, and mucosal wound model data from colon; () = reported cellular source.

*WBI* whole-body irradiation, *MP* macrophage, *MyoF* myofibroblast, *DC* dendritic cell, *DSS* dextran sulfate sodium, *TNBS* trinitrobenzene sulfonic acid, *NA* not applicable.

Multiple lines of evidence suggest a key functional role of Wnt/β-catenin signaling during intestinal regeneration. Mice carrying reduced expression of *Dickkopf-related protein (Dkk)1*, a Wnt/β-catenin antagonist, show faster recovery from DSS-induced colitis [104]. Furthermore, deletion of Wnt receptors *Fzd2* and *Fzd7* from intestinal Lgr5^+^ cells impaired regeneration upon irradiation [105]. In another study, apoptotic cell death of Lgr5^+^ cells at 24 h after a lethal dose of irradiation in mice was rescued pharmacologically in vivo by treatment with a novel Wnt-activating small compound [89]. These findings were validated in vitro in mouse intestinal and human colon organoids, where Wnt stimulation rescued crypt formation and Wnt target gene expression upon irradiation with 8 Gy [89].

In support of an early role of Wnts in driving the regenerative response, mouse intestinal organoids that were cultured in a collagen type I-containing matrix and, at the same time, supplemented with Wnt3a displayed a downregulation of classical Wnt stem cell genes and differentiation markers, and upregulation of fetal markers, including *Sca-1*, *Anxa1*, and *Trop2* [75]. Thus, extracellular matrix composition together with a Wnt-stimulus may directly promote the regenerative epithelial response. Furthermore, key Wnts may be produced during regeneration by both mesenchymal and epithelial sources. At 2 days after irradiation, an increase in *Wnt2b* expression was detected in both Ng2^+^ pericyte-like and epithelial cells [24]. Blocking Wnt secretion in Ng2^+^ cells, that display radio-resistance and proliferate during injury, impaired regeneration. The increase in Wnt expression in stromal Ng2^+^ cells involved Shh-signaling, as the Gli2 transcription activator bound the *Wnt2b* and *Wnt9b* promoter regions in CHIP-Seq data [24]. Another study reported that irradiation induced the expression of *Wnt2b* at 1 day after irradiation in Tert^+^ epithelial cells, long-lived reserve stem cells located at +3/+4 position that exit a quiescent state upon injury to drive regeneration [78]. Blocking *Wnt2b* expression using shRNAs prevented growth of irradiated organoids. Furthermore, the *Wnt2b* promoter was found to harbor multiple hypoxia-inducible factor-response elements, linking Wnt expression to hypoxic injury [78]. Together, these findings suggest that both mesenchymal and epithelial Wnts promote the mobilization of reserve stem cells to perform the regenerative epithelial response. Of note, Wnt-independent routes to β-catenin activation may also be considered, as shown in PGE2-treated organoids, that adopt a WAE state and display nuclear β-catenin localization [99]. In this case, the proposed mechanism of β-catenin activation involves PGE2 receptor-induced protein kinase A (PKA)-mediated inhibition of GSK3β rather than direct stimulation with Wnt ligands [99].

Additional roles of β-catenin-independent Wnt pathways were uncovered in various intestinal injury models, as shown by increased levels of Wnt5a (Table 1). During colon homeostasis, *Wnt5a*-expressing cells are mainly found at the upper regions of colon crypts, while wounding induces an accumulation of *Wnt5a*-expressing stromal cells at the crypt base, near the regenerating epithelium [72]. *Wnt5a* expression was found responsible for the formation of invaginations called wound channels in the epithelium in vivo, as well as in organoids in vitro, using Wnt5a-coated beads. A model is presented in which *Wnt5a* expression locally induces TGFβ signaling to reduce epithelial proliferation, which promotes crypt regeneration to restore homeostasis [72]. Furthermore, increased *Wnt5a* expression in fibroblasts upon DSS-induced injury may promote inflammation [101]. Deletion of *Wnt5a* suppressed pro-inflammatory cytokine production by dendritic cells and tissue damage, suggesting that Wnt5a contributes to the inflammatory response. Lastly, mesenchyme-derived Wnt5a may be involved in the direct activation of YAP signaling, while antagonizing Wnt/β-catenin signaling [96]. Thus, mesenchymal-derived Wnt5a seems to affect both the inflammatory response as well as epithelial remodeling during regeneration.

### RSPO-LGR4 signaling cooperates with Wnts during intestinal regeneration

Multiple studies revealed a positive effect of RSPOs on the intestinal epithelial repair after chemical- and irradiation-induced damage, further supporting a key role of Wnt signaling during injury repair. Injury-induced RSPO expression was mainly found in the mesenchymal compartment (Table 1). In DSS-induced colitis models, deletion of *Rspo3* from PDGFRα^+^ or Myh11^+^ mesenchymal cells reduced epithelial regeneration [25, 102]. Moreover, recombinant hRSPO1 injection promoted mucosal regeneration in models of DSS- or TNBS-induced colitis [106], and adenovirus-mediated delivery of hRSPO1 protected mice from 10.4 Gy whole-body irradiation and improved mortality rates [107]. By contrast, upon antibody-mediated blockade of RSPO2 and RSPO3, irradiation-induced regeneration was impaired [108].

Various lines of evidence indicate that Lgr4 is the primary receptor used by RSPO proteins to drive regeneration. First, while *Lgr5* expression is lost upon DSS- or radiation-induced injury, *Lgr4* remains expressed in differentiated cells outside crypt areas, according to fluorescence in situ hybridization and single-cell RNA-sequencing data [102, 109]. Furthermore, *Lgr4*-mutant mice are highly susceptible for DSS-induced damage, which is alleviated when downstream Wnt/β-catenin signaling is activated simultaneously via *APC* mutations or pharmacological GSK3β inhibition [110]. Together, these findings thus suggest that RSPO-Lgr4 signaling is a key event during injury and repair. In current models, key targets of RSPO-LGR signaling are the membrane E3 ligases Rnf43 and Znrf3 [28, 111], although both comprise Wnt target genes that are downregulated during regeneration. Thus, if and how RSPO-mediated signaling routes operate precisely during regeneration remains an interesting subject for future investigation.

## Conclusions and future perspectives

Recent advances in the profiling of the regenerative intestinal epithelium combined with methods of lineage tracing and organoid-based applications have uncovered a remarkable adaptability of the intestine. Upon tissue damage, various cell types can convert into a transient fetal-like proliferative state to replace lost stem cells, promote tissue remodeling, and ultimately restore homeostasis. Indeed, multiple parallels can be drawn between intestinal development and intestinal damage repair, including by (1) an overlap in transcriptional signatures, (2) a shared pattern of uniform proliferation, (3) a decreased expression of adult stem cell markers that depend on Wnt/RSPO-high domains, (4) the possibility to generate stably propagated spheroid cultures that maintain fetal signatures and lack budding structures in vitro, and (5) the involvement of β-catenin-independent Wnt pathways for tissue patterning in vivo. Together, these insights have changed the traditional view on intestinal cell fate determination, shifting from a hierarchical organization to a more dynamic model in which cells retain the ability to switch states depending on tissue context and microenvironment. Strikingly, although fetal conversion comprises a transient state during tissue regeneration in vivo, this state can be captured and stably propagated when cells are cultured using organoid-based methods in vitro. These findings indicate that signals derived from the microenvironment, e.g. from mesenchymal cells, immune cells, and extracellular matrix, are essential to guide the transition from fetal to homeostatic restoration. The recent advances in in vitro model systems that recapitulate cycles of injury and repair will be of great value in gaining an in-depth understanding of the underlying molecular cell–cell communication events.

A number of poorly understood issues await further investigation. What damage signals initiate fetal conversion of the epithelium? And, as fetal signatures in different model systems only show partial overlap, how do fetal programs correlate with type of injury, reverted cell-of-origin or timing of analysis? Furthermore, despite the downregulation of Wnt target genes during regeneration, whether regenerative spheroids grow in a Wnt/RSPO-independent manner remains inconclusive. Besides, Wnt ligand production in both epithelial, mesenchymal, and immune cells at early stages of tissue damage appears essential for efficient tissue repair. What is the role of β-catenin-independent Wnt signaling during early stages of intestinal regeneration? How does the interplay between YAP and Wnt signaling guide the reestablishment of tissue homeostasis? Regarding the transient nature of the involved key signaling events, solving the underlying mechanisms will likely depend on advanced methods of single-cell analysis and live imaging. Ultimately, improved insight in the regulation of intestinal plasticity may fuel the design of novel therapeutic approaches that aim to restore intestinal barrier function, for instance upon chemotherapeutical or radiation treatment in cancer, or in patients suffering from gastrointestinal disease.
